# The National Hospital for the Paralysed and Epileptic

**Published:** 1889-07-13

**Authors:** 


### The Hospital Workshop.

### No. V.—THE NATIONAL HOSPITAL FOR THE PARALYSED AND EPILEPTIC.

### Out-Patients.

There are good and bad special hospitals: bad in the sense of being proprietary concerns run for private ends, and for the treatment of diseases already provided for by the general institutions. Cancer, for instance, may be taken as the type of an affection that should be left to the ordinary operating surgeon. But there are other special hospitals that have done, and are still doing, work of no little importance. They have forced the hand of the medical schools, so that each one now possesses its own group of specialists, and by advancing the scientific study of their subjects they have amply proved their right to a separate existence. Moreover, by a system of pluralities, hospital appointments lie in the hands of the few, and often the only field that lies open to strong individuality is in these special institutions. Many men seem to have recognised the fact that the general practitioner who would attain to name and fame must carve his own way to fortune as a free lance.

The National comes in the front rank of the good special hospitals. It affords an amount of relief to suffering humanity that can hardly be over-estimated, and its doors are opened to many chronic and obscure cases that could not be received by other charities. As a centre for scientific teaching, its advantages are unequalled. An immense collection of nervous diseases of every degree of variety is here seen under the care of men whose names enjoy a world-wide renown. What member of the medical profession would not kindle with a glow of pride in his great and progressive science at the mention of the names of David Ferrier, Hughlings Jackson, Gowers, Charlton Bastian, Anderson, and Victor Horsley? Of such a distinguished knot of men any hospital in Europe might well be proud. The practice of the hospital is at present free to all qualified men, but it is intended to organise a more thorough course of training, for which entrance fees will probably be charged.

The new building at Queen-square is of the modern order, very different from the plain, old-fashioned, and time-worn hospitals, such as Guy's and the London. Here are lofty halls and corridors with mosaic floors, plate-glass windows, marble pillars, and many beauties of architecture. The out-patient room is large, light, and comfortable. Attendances average about 23,000 yearly, with 3,000 more for electrical treatment. Two physicians sit at the same time, and new comers are seen first. A long time is thus taken up, for diseases of this kind cannot be hurried over, and it is generally five o'clock before the old cases are begun. This is undoubtedly a great disadvantage to the patients, but one hardly knows how the difficulty is to be surmounted. Some help might be got by appointing more assistant physicians to whom the seniors could hand over all the less important cases.

Your commissioner visited the out-patient room one Friday afternoon and was fortunate in being introduced to Dr. Ferrier, whose localisation of brain centres has rendered his name famous and at the same time laid the foundation of modern brain surgery. Briefly, he has shown that certain parts of the grey matter on the surface of the brain are concerned in producing definite muscular movements. Thus, there is a centre for the leg, a second for the arm, others for the face, the tongue, and so on. When one of these spots is irritated, as by a tumour, it is sometimes possible from the symptoms to point out to the surgeon exactly where to raise a piece of the skull and find the growth. This branch of surgery is still in its youth, but brilliant results have been already obtained, and there can be little question that a great field of future conquest has been opened up by the painstaking researches and genius of Ferrier.

There is a distinctiveness about the patients. Not a few are dark, gaunt, tremulous, of a markedly nervous temperament. Some have a curious look about the eyes, or the lines of the face are blurred, the eyelips droop, the mouth is drawn, the jaw fallen, limbs are fixed or helpless from various palsies. There are many odd gaits, the shuffle of paralysis where the toe is pushed along the ground, or the high step of locomotor ataxy, when the legs are thrown out toes in the air, while the patient has to watch his footsteps carefully. Some hobble in on crutches, or with the help of a stick; a few are seated in wheel-chairs, others walk alone, or lean feebly on the arm of a friend.

The first patient who appears is a red-cheeked countryman. He complains of pains in his head for some years past, worse during the last thirteen months, and also of dimness of sight. His left leg has been getting weaker and weaker. Then follow a number of tests of nerve-power. The patient is told to walk to the other end of the room and back again, which he does fairly well. He is made to stand with heels close together and eyes shut to find out if his muscles act in concert. In order to compare the strength of his arms, he is directed to squeeze a kind of oval spring, with a registering dial, first in one hand and then in the other. This gives pretty normal and equal results, and proves that the loss of power does not affect the upper limbs. The legs are tested in turn by fixing the heel on the ground and raising the toes in the air, and the left is found to be much the weaker. The face muscles are thrown into action by showing the teeth in a forced grin, and it is at once evident both sides of the face do not act equally well. Muscular tremor is detected by the patient holding out both hands with the backs upwards. What are called the "reflexes" are then tried. Every schoolboy knows that when the thighs are loosely crossed, and the tendon just below the knee is smartly tapped, the leg kicks out of its own accord. In certain spinal diseases, this "knee-jerk," as it is called, is either overdone, or may be entirely absent; in fact, it becomes a valuable test of the proper working of the nervous mechanism. There are many other reflexes in other parts of the body. After their examination, the head is tapped with the fingers, and found to be almost everywhere tender.

In this case—as in most others—the eyes are carefully examined. The pupils or "apples" may be enlarged, or contracted to the size of a pinhole, or fixed, or unequal, all of which symptoms are of value. The inside of the eyeball is seen by means of a perforated mirror, the "ophthalmoscope." In the patient before us, the white spots, known as the "discs," where the optic nerves enter the eye, have become dwindled or "atrophied."

Now this man's condition is a serious one. He is thirty years of age only, and without treatment would go on from bad to worse until he became blind, palsied and possibly insane. Under proper care, however, he will recover almost, if not quite entirely. A cure of this sort, therefore, saves the community from the burden of his future support, a direct gain to the State which has lately been insisted on by Sir James Paget.

To the question "What are you complaining of?" the next patient answers pithily "Fits." He is a young man, and until lately acted as clerk in the meat-market at Smithfield, but was discharged from his situation after having had several fits in the desk. Unfortunately, that history is the type of a large number of cases where the uncertainty of epileptic seizures keeps those affected out of employment.

A boy is carried in by a woman, who says she is the aunt, and he has been sent in from the country. He is doubled up, both eyes squinting outwards, head fixed, and has been unable to walk for the last six weeks. The poor child is deaf in the right ear, is paralysed on the same side of the face and on the opposite side of the body, and can speak only very indistinctly. Mr. Ferrier inclines to the opinion that it is tubercular disease, points out the parts of the brain affected, and sends on the child for admission to the wards.

A good-looking man of thirty-three hobbles in, leaning on a stick. One half of the body is paralysed, the right leg is dragged along stiffly, while the arm of the same side is bent across the body, the thumb being tightly clenched between the fingers. The case is overhauled point by point. Neither face nor speech are affected. One curious fact is that although he has no power over the right foot, yet he can show exactly by moving the other foot in what position the palsied one is placed, whether bent upwards or downwards, in or out. That remarkable faculty is called the "muscular" sense, and is present although the limb is utterly powerless. It is sometimes described as the sixth sense. The patient's history and symptoms show that the mischief began in a bit of the patient's brain known as the "leg-centre," and, spreading thence, has involved the neighbouring "arm-centre." One is glad to hear that cure is not hopeless and that physic will most likely work wonders, although much valuable time has been lost.

The next patient has a queer glassy look about the eyes, due to the fact of his pupils being fixed and of pinhole size. He is forty-nine years of age, and is alike jerky in speech, manner, and walk. It does not take long to find out he is suffering from "locomotor ataxy," a disease in which a portion of the spinal cord is affected, and the sufferer gradually loses the power of making his muscles work together, so that all his movements become abrupt and uncertain. The shooting or "lightning" pains often present are almost always put down to rheumatism, and our patient informs us that his illness began three years back with what the doctors certified as "rheumatic catarrh." He cannot stand upright with eyes shut and heels together, but sways about and would fall unless caught. He is obliged to watch every step, and throws his feet out with the toes pointed upwards. He states that he is very nervous about the street crossings. That is a common symptom, for, owing to the muscular uncertainty, the patient loses confidence in his powers. The "knee-jerk" is absent. There is no "girdle pain," or feeling as of a cord tied round the body, a condition commonly met with in this disease. Locomotor ataxy, which has been discovered of late years only, may run a very chronic course. As yet treatment has been of little avail; but quite lately the novel and somewhat startling plan of "suspension" has been much practised. The patient is repeatedly lifted from the ground for a short period by pulleys fastened to the head, and the spinal cord is thereby stretched. Dr. Ferrier's casual remark, "I am going to hang this man," caused some evident alarm to the friend who accompanied him, until reassured by the doctor's kindly manner.

A painful object is the next patient, a boy of uncertain age, brought in by his mother, who says there is something wrong with "the spine of his back." He is a white-faced lad, with prominent eyes, and the head projects backwards in an extraordinary "boot shape." The lower limbs are twisted with rickets; little more than skin and bone; quite useless and wasted; and blue with congestion. The child has plainly been the subject of infantile paralysis, a disease in which instant and active treatment alone can be of any avail. The mother is accordingly told that nothing can now be done for her child. She is advised to get a chair for him, and, when she says she cannot afford to buy one, is directed to the Samaritan Fund.

The out-patient room is all one could wish, and its arrangements are excellent. The National Hospital for the Paralysed and Epileptic is carrying on a work full of strong, vivid human interest; and here one may witness a great scientific warfare which is being waged against some of the most obscure and terrible diseases which attack mankind.

